# Supplementary material for: Nutrition Knowledge and Food Literacy Among Persons with Multiple Sclerosis—Development and Validation of Patient-Reported Outcome Measures
Source: Nutrients. 2024 Nov 26;16(23):4043. doi: 10.3390/nu16234043 (PMC11643976; doi:10.3390/nu16234043)
Supplement: Supplementary file 1 [file nutrients-16-04043-s001.zip › nutrients-3305940-supplementary.pdf]

Table S1: The items of the SFLQ and the corresponding MSFL items used for the cognitive debriefing

**SFLQ items**	**MSFL items for cognitive debriefing**
When I have questions on healthy nutrition, I know where I can find information on this issue.	If I have queries about nutrition for MS, I know where to find reliable information.
In general, how well do you understand the following types of nutritional information? (What is meant is the comprehensibility and not the quality of the information.)<br>(A) Nutrition information leaflets<br>(B) Food label information<br>(C) TV or radio program on nutrition<br>(D) Oral recommendations regarding nutrition from professionals.<br>(E) Nutrition advice from family members or friends	In general, how well do you understand the following information about nutrition for MS? (What is meant is the comprehensibility and not the quality of the information.)<br><br>Please tick one answer for each line.<br><br>• Information in brochures or books<br>• Information on websites or in podcasts<br>• Verbal recommendations from healthcare professionals
How familiar are you with the Swiss Food Pyramid?	How familiar are you with the recommendations for healthy eating for people with MS?
I know the official Swiss recommendations about fruit and vegetable intake.	n.a.
I know the official Swiss recommendations about salt intake.	n.a.
Think about a usual day: how easy or difficult is it for you to compose a balanced meal at home?	- It's easy for me to prepare a vegetarian main meal.<br>- In everyday life, I find it easy to maintain a predominantly plant-based diet.
In the past, how often were you able to help your family members or a friend if they had questions concerning nutritional issues?	- How easy is it for you to exchange ideas with others about nutrition for MS?<br>- I feel confident to talk to others about nutrition for people with MS.<br>- I feel confident to discuss nutritional recommendations for people with MS with others.
There is a lot of information available on healthy nutrition today. How well do you manage to choose the information relevant to you?	- How well do you manage to select the information that is relevant to you about healthy eating for MS?<br>- How well do you manage to distinguish important information about nutrition for MS from not so relevant information?
How easy is it for you to judge if media information on nutritional issues can be trusted?	How easy is it for you to judge whether information about nutrition for MS is trustworthy?
Commercials often relate foods with health. How easy is it for you to judge if the presented associations are appropriate or not?	On the Internet, individual foods are often associated with an improvement or a deterioration in the course of MS. How easy is it for you to judge to what extent the relationships presented are true or not?
How easy is it for you to evaluate if a specific food is relevant for a healthy diet?	How easy is it for you to assess the role that different food groups play in a health-promoting diet for MS?
How easy is it for you to evaluate the longer-term impact of your dietary habits on your health?	How easy is it for you to assess what impact your eating habits could have on your MS in the long term?

n.a.= not applicable

Table S2: The items of the final MSFLQ

1.	If I have queries about nutrition for MS, I know where to find reliable information.
2.	In general, how well do you understand the following information about nutrition for MS? (What is meant is the comprehensibility and not the quality of the information.)<br><br>Please tick one answer for each line.<br><br>• Information in brochures or books<br>• Information on websites or in podcasts<br>• Verbal information from healthcare professionals
3.	How familiar are you with the recommendations for healthy eating for people with MS?
4.	How well do you manage to select the information that is relevant to you about healthy eating for MS?
5.	How easy is it for you to judge whether information about nutrition for MS is trustworthy?
6.	How well do you manage to distinguish relevant information about nutrition for MS from not so relevant information?
7.	On the Internet, individual foods (e.g. cow's milk, pork or chia seeds) are often associated with an improvement or a deterioration in the course of MS. How easy is it for you to judge to what extent the relationships presented are true or not?
8.	How easy is it for you to assess the role that different food groups (e.g. vegetables, fish or meat) play in a healthy diet for MS?
9.	How easy is it for you to assess what impact your eating habits could have on your MS in the long term?
10.	It's easy for me to prepare a vegetarian main meal.
11.	In everyday life, it's easy for me to eat significantly more plant-based foods than animal-based foods.
12.	How easy is it for you to exchange ideas with others about nutrition for MS?

Table S3: The items of the final MSNKQ

1. People with MS should follow a predominantly plant-based diet with lots of vegetables, fruits and pulses and avoid cow's milk and dairy products. (false)
2. Consumption of cow's milk and dairy products should be avoided if you suffer from MS. (false)
3. The consumption of foods containing gluten (e.g. bread or pasta) should be avoided if you have MS. (false)
4. Individual foods (e.g. cow's milk, pork or chia seeds) can have an influence on the course of MS. (false)
5. People with MS should take B vitamins, zinc, and selenium regularly. (false)
6. Taking high doses (> 4000 international units/day) of Vitamin D has a positive effect on the course of MS. (false)
7. In people who are extremely overweight (obese), the vitamin D level in the blood is often too low. (true)
8. Being obese has a detrimental impact on the course of MS. (true)
9. People with MS are recommended to eat fish 1-2 times a week, but "fatty fish" such as herring, salmon and mackerel should be avoided. (false)
10. Taking Omega-3 fatty acid capsules has a beneficial effect on the course of MS. (false)
11. A diet that reduces the risk of cardiovascular disease can also have a positive impact on the course of MS. (true)

Table S4: Feedback from participants on the MSNKQ and the MSFLQ

	MSNKQ				MSFLQ			
	Disagree (%)		Agree (%)		Disagree (%)		Agree (%)	
	1	2	3	4	1	2	3	4
Filling out the questionnaire made me curious to look (more) into the topic of nutrition and MS.	10.1%	15.5 %	39.2%	35.1%	11.5%	14.2%	45.3%	29.1%
I was irritated by many of the questions asked.	39.2%	31.1 %	24.3%	5.4%	45.3%	36.5%	16.9%	1.4%
I found it difficult to answer many of the questions because I was lacking the necessary knowledge.	21.6%	27.0 %	39.2%	12.2%				
Many of the questions were difficult to understand.	70.3%	19.6 %	9.5%	0.7%	55.4%	35.8%	8.1%	0.7%
Filling out the questionnaire was stressful.	88.4%	9.5%	2.0%	0%	85.7%	10.9%	3.4%	0%
Filling out the questionnaire took too long.	91.2%	7.4%	1.4%	0%	78.4%	15.5%	5.4%	0.7%
